# Differentiation of the Endometrial Macrophage during Pregnancy in the Cow

**DOI:** 10.1371/journal.pone.0013213

**Published:** 2010-10-07

**Authors:** Lilian J. Oliveira, Steve McClellan, Peter J. Hansen

**Affiliations:** 1 Department of Animal Sciences and D.H. Barron Reproductive and Perinatal Biology Research Program, Interdisciplinary Center for Biotechnology Research, University of Florida, Gainesville, Florida, United States of America; 2 Flow Cytometry Core Laboratory, Interdisciplinary Center for Biotechnology Research, University of Florida, Gainesville, Florida, United States of America; University of Toronto, Canada

## Abstract

**Background:**

The presence of conceptus alloantigens necessitates changes in maternal immune function. One player in this process may be the macrophage. In the cow, there is large-scale recruitment of macrophages expressing CD68 and CD14 to the uterine endometrium during pregnancy.

**Methodology/Principal Findings:**

In the present study, the function of endometrial macrophages during pregnancy was inferred by comparison of the transcriptome of endometrial CD14^+^ cells isolated from pregnant cows as compared to that of blood CD14^+^ cells. The pattern of gene expression was largely similar for CD14^+^ cells from both sources, suggesting that cells from both tissues are from the monocyte/macrophage lineage. A total of 1,364 unique genes were differentially expressed, with 680 genes upregulated in endometrial CD14^+^ cells as compared to blood CD14^+^ cells and with 674 genes downregulated in endometrial CD14^+^ cells as compared to blood CD14^+^ cells. Twelve genes characteristic of M2 activated macrophages (*SLCO2B1*, *GATM*, *MRC1*, *ALDH1A1*, *PTGS1*, *RNASE6*, *CLEC7A, DPEP2*, *CD163*, *CCL22*, *CCL24*, and *CDH1*) were upregulated in endometrial CD14^+^ cells. M2 macrophages play roles in immune regulation, tissue remodeling, angiogenesis and apoptosis. Consistent with a role in tissue remodeling, there was over-representation of differentially expressed genes in endometrium for three ontologies related to proteolysis. A role in apoptosis is suggested by the observation that the most overrepresented gene in endometrial CD14^+^ cells was *GZMA*.

**Conclusions:**

Results indicate that at least a subpopulation of endometrial macrophages cells differentiates along an M2 activation pathway during pregnancy and that the cells are likely to play roles in immune regulation, tissue remodeling, angiogenesis, and apoptosis.

## Introduction

Pregnancy leads to immunological adjustments in the mother that facilitate survival of the conceptus. One of these adjustments, recruitment of macrophages to the pregnant endometrium, occurs in a wide range of mammalian species including the mouse [1], human [2]–[4], cynomologus and vervet monkeys [5], sheep [6] and cow [7], [8]. The role of endometrial macrophages is not known and may vary depending upon anatomical location. In cattle, macrophages are rare in the endometrium in nonpregnant females but accumulate in large number during pregnancy [7]. Endometrial macrophages in the pregnant cow are regionally differentiated, with macrophages in the interplacentomal endometrium being largely CD68^+^CD14^+^MHC class II^-^ and often positive for CD11b while the less-numerous macrophages in the caruncular septa of placentomes are largely CD68^+^CD14^+^MHC class II^+^ and have little or no expression of CD11b [8].

One role for endometrial macrophages is participation in host defense. Indeed, endometrial macrophages increase in number in the pregnant cow after experimental infection with *Neospora caninum* [9]. A role for macrophages in clearing bacteria after parturition or in the expulsion of the conceptus is also a possibility because macrophages accumulate with impending parturition [10], [11]. Activation of a local immune response in response to intrauterine infection can also turn macrophages into pregnancy foes that induce fetal resorption [12]. Endometrial macrophages in the human have been proposed to participate in vascular remodeling since they produce vascular endothelial growth factor [13] and macrophage accumulation in the uterus is abnormal in women with pre-eclampsia [14]. Macrophages can also promote trophoblast function by clearing apoptotic cells and secreting cytokines that regulate the apoptotic process [15]. In addition, macrophages can metabolize placental lactogen [16] and may participate in regulation of concentrations of this hormone at the fetal-maternal interface. Given the potential antigenicity of the conceptus by virtue of inheritance of paternal antigens and, at least in cows, the expression of classical MHC proteins on the placenta [17], [18], a role for macrophages in limiting activation of anti-conceptus immune responses is also possible.

Macrophages can follow different differentiation pathways when activated by cytokines. Exposure to interferon-γ (IFNG), tumor necrosis factor-α and/or lipopolysaccharide leads to activation along the classical or M1 activation pathway to produce a macrophage that promotes inflammation and cytotoxicity while exposure to interleukin-4 (IL4) and -13 (IL13) leads to activation via the alternative or M2 activation pathway to produce a macrophage that directs immunosuppression and wound healing [19], [20]. The M2 activation pathway can be divided into three subpathways. The M2a pathway represents macrophages that are stimulated by IL13 and IL4; the M2b pathway involves macrophage differentiation under the influence of immune complexes via toll-like receptor and IL1 receptor; and M2c macrophages differentiate under the influence of IL10 and transforming growth factor-β (TGFB) or glucocorticoid hormones [20]. Endometrial macrophages in humans and mice have been reported to have some characteristics of M2 activation [2], [4], [21]. It remains possible that endometrial macrophages possess a unique activation status.

In the experiment reported herein, likely functions of macrophages residing in the interplacentomal endometrium of pregnant cows were determined through use of global analysis of the macrophage transcriptome to characterize the pattern of gene expression and identify functional pathways that are activated or inhibited in endometrial macrophages compared with monocytes in blood. We hypothesized that macrophages in the pregnant endometrium would express genes that support placental growth (angiogenesis and tissue remodeling) and that are indicative of a M2 phenotype that signifies a role in modulation of immune responses towards fetal antigens.

## Results

### Purification of Endometrial and Blood CD14^+^ Cells

**Figure 1:**
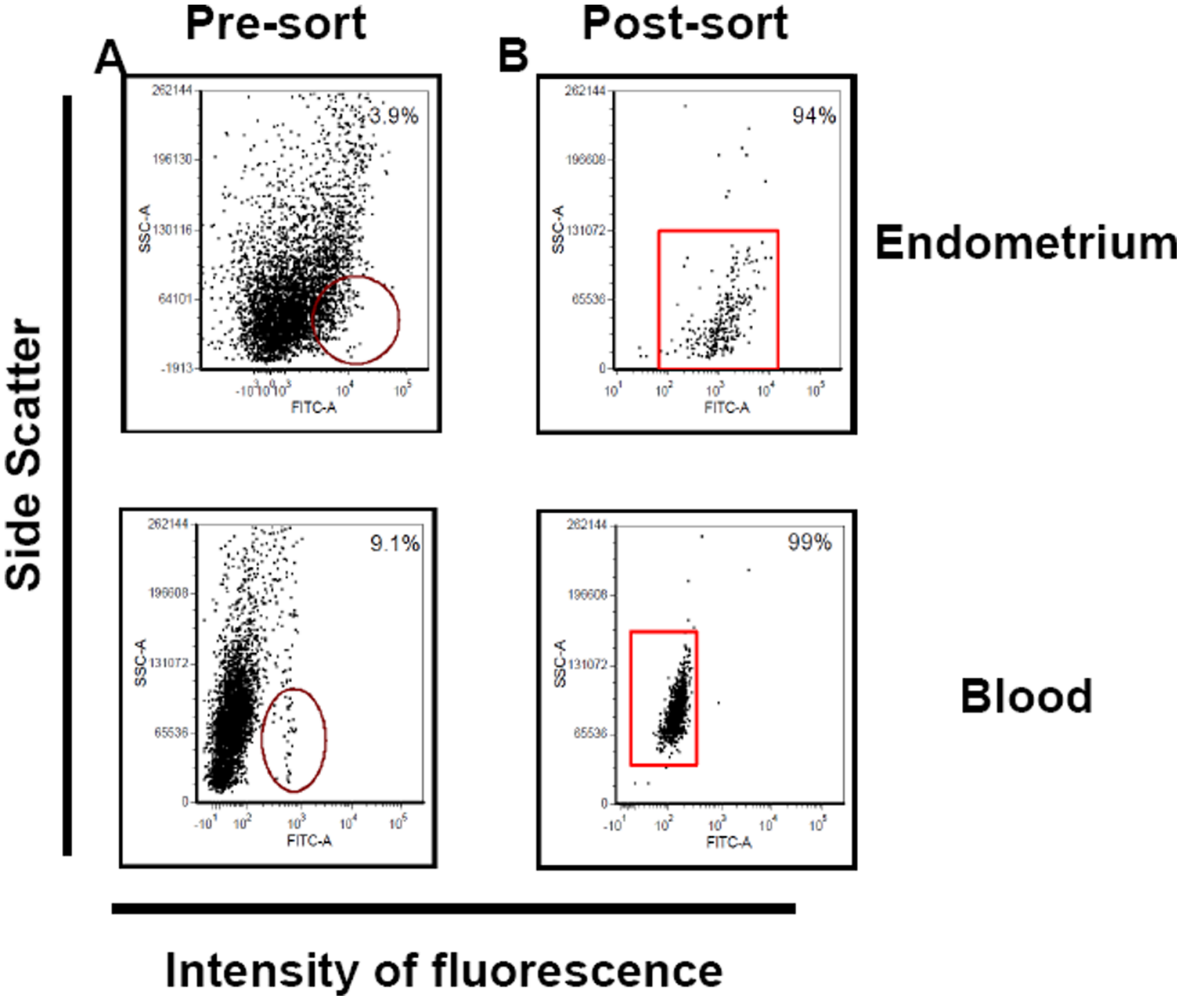
Representative acquisition dot plots for CD14^+^ cells in endometrium and peripheral blood as determined by flow cytometry. Shown are analyses for dispersed endometrial cells and peripheral blood mononuclear cells from a cow at ∼Day 230 of pregnancy before (A) and after two rounds of cell sorting (B). The y-axis depicts side scatter characteristics (SSC) to analyze cells on the basis of granularity and the x-axis depicts intensity of fluorescence (FL1) associated with anti CD14. The circles and rectangles in the dot plots identify CD14^+^ cells targeted for sorting.

The final preparation of endometrial CD14^+^ cells had a purity of 90–95% based on flow cytometry while blood CD14^+^ cells were 98–99% pure (Figure 1).

### Expression of Genes in Blood and Endometrial CD14^+^ Cells

**Figure 2:**
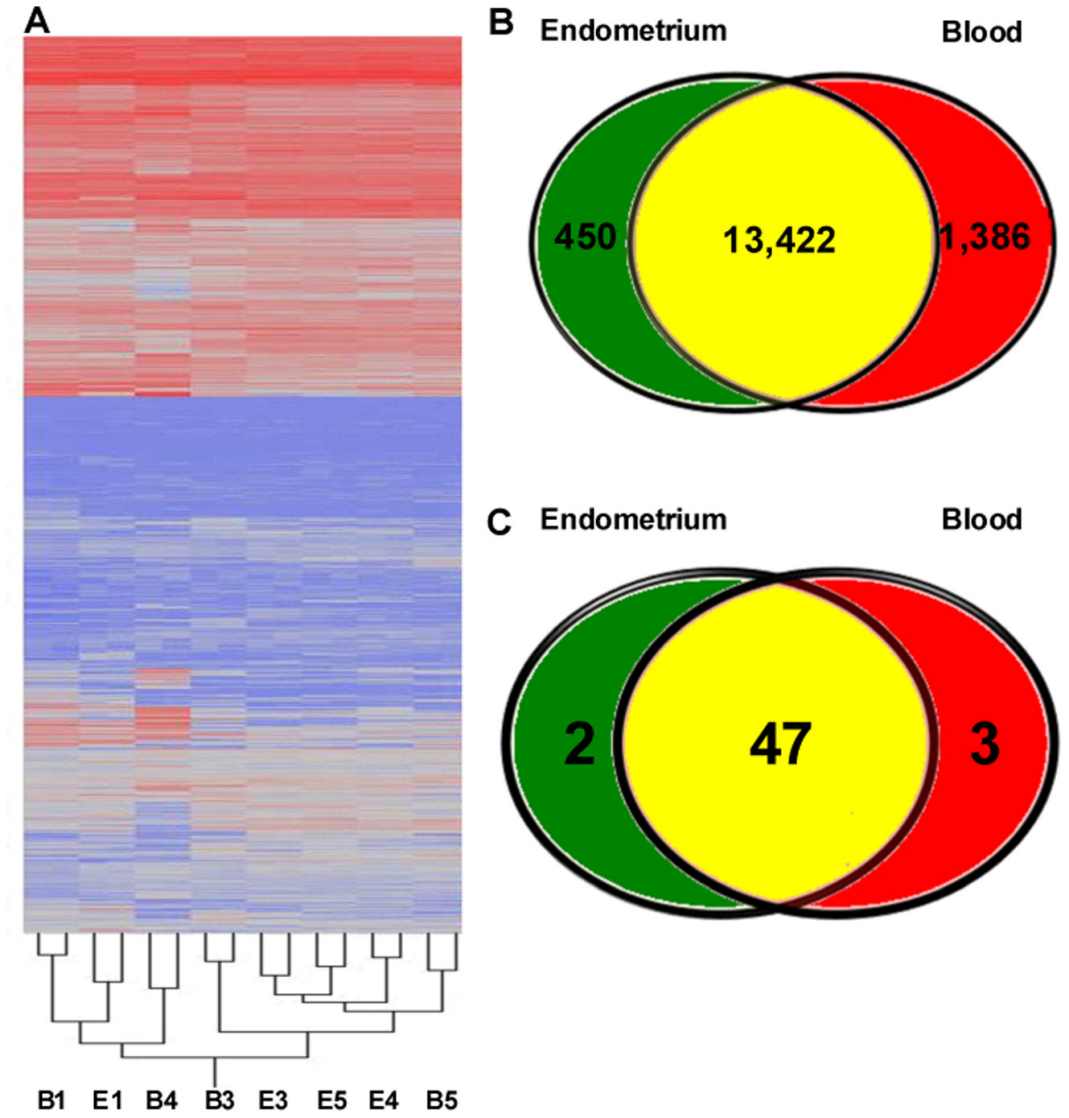
Graphical representation of differentially regulated genes in endometrial and blood CD14+ cells. (A) Hierarchical cluster representing the global gene expression of endometrial and blood CD14^+^ cells. Each sample is identified by tissue source [endometrium (E) and blood (B)], dye (Cy3 vs Cy5), replicate (A vs M), and cow number (1 to 5). (B) Venn diagram characterizing expressed genes in endometrium and blood. Circles represent the genes expressed in both cell types (brown; 13,422 genes), genes exclusively expressed by endometrial CD14^+^ cells (green; 450 genes) and genes expressed exclusively by blood CD14^+^ cells (red; 1386 genes). (C) Venn diagram characterizing the expression of immune genes reported by Jensen et al. [22] to be expressed by the bovine macrophages. Circles represent the genes expressed in both cell types (brown; 47 genes), genes exclusively expressed by endometrial CD14^+^ cells (green; 2 gene) and genes expressed exclusively by blood CD14^+^ cells (red; 3 genes). An additional 4 genes reported by Jensen et al. [22] were not identified in either cell population.

The transcriptomes of blood and endometrial CD14^+^ cells were compared using a bovine whole genome array that covers ∼19,500 bovine genes. As shown from results of hierarchical cluster analysis (Figure 2A), the pattern of gene expression was largely similar for CD14^+^ cells from both sources, suggesting that cells from both tissues are from the monocyte/macrophage lineage. There were 13,422 genes expressed in both cell types, 450 genes exclusively expressed by endometrial CD14^+^ cells and 1,386 genes expressed exclusively by blood CD14^+^ cells. (Figure 2B).

### Identification of Differentially Expressed Genes

Criteria for identifying differentially expressed genes was a level of significance of P≤0.05, a fold change >2, and a maximum false discovery rate of 0.01. A total of 1,364 unique genes were differentially expressed, with 680 genes upregulated in endometrial CD14^+^ cells as compared to blood CD14^+^ cells and with 674 genes downregulated in endometrial CD14^+^ cells as compared to blood CD14^+^ cells (i.e, upregulated in blood). Of these differentially expressed genes, 598 of the 680 upregulated genes in the endometrium and 529 of the 674 upregulated in the blood could be annotated. A list of the differentially-expressed genes, including least-squares means for endometrium and blood, fold-change values and P values is presented in File S1.

The gene with the greatest fold-increase (120.2 fold) in the endometrium was granzyme A (*gzma;* NM_001001142), a serine proteinase enzyme produced by cytotoxic T cells and natural killer cells, which induces apoptosis of target cells. Transcripts for a second granzyme A gene, (*GZMA*, NM_001099095) was increased in endometrium by 5.8 fold in endometrial CD14^+^ cells while another granzyme gene, *GZMB*, was upregulated 22.5 fold. There were other genes characteristically expressed in immune cells that were among the top 100 overexpressed genes such as stanniocalcin-1 (*STC1*; 110.4 fold), heat shock protein70 B (*HSPA6*; 55.6-fold) and macrophage scavenger receptor A (*MSR1;* 7.2 fold).

Several growth factor and cytokine genes were upregulated in endometrium (File S1). These include connective tissue growth factor (*CTGF;*39.3 fold), insulin-like growth factor binding protein-6 (*IGFBP6;* 18.0 fold), endothelial cell-specific molecule 1 (ESM; 46.9 fold), chemokine (C-X-C motif) ligand 14 (*CXCL14;* 36.7 fold), and chemokine (C-C motif) ligand 22 (*CCL22*; 29.0 fold). Additionally, there were molecules involved in complement system regulation [V-set and immunglobulin domain-containing 4 (*VSIG4;* 20.4 fold), clusterin (*CLU*; 27.1 fold), and complement component (*C1QA*; 19.1 fold)], genes related to cell adhesion [nectin-3 (*PVRL3*; 19.5 fold), claudin-1 (*CLDN1*; 28.1 fold), and claudin-8 (*CLDN8*; 44.6 fold)], and genes involved in responses to hypoxia [aryl-hydrocarbon receptor nuclear translocator 2 (*ARNT2*; 22.4 fold), angiogenin (*ANG*; 38.1 fold) and L-arginine:glycine amidinotransferase (*GATM;* 30.8 fold)].

Several genes characteristically produced by the placenta were upregulated in endometrial CD14^+^ cells (File S1). These genes include several members of the pregnancy-associated glycoprotein family (in order of fold change, *PAG3*, *PAG6*, *PAG15*, *PAG21*, *PAG5*, *PAG1B*, *PAG4*, *PAG10*, *PAG7*, *PAG17*, *PAG11*, *PAG9*, *PAG18*; 72.2 to 3.6 fold increase), placental lactogen (*CSH1*; 43.8 fold), and a group of genes encoding prolactin-related proteins (*PRP3*, *PRP6*, *PRP15*, *PRP1*, *PRP8*; 54.3 to 30.7 fold increase). Uterine milk protein precursor, a product of uterine epithelium also called uterine serpin (*SERPINA14*), was also highly expressed in endometrial CD14^+^ cells (92.5 fold higher than blood). Thus, it is likely that some cells in the endometrial CD14^+^ cell preparation were of placental and endometrial epithelial origin.

Calcitonin-related polypeptide β, transcript variant 2 (*CALCB*) was the gene most upregulated in blood CD14^+^ cells (23.8 fold higher in blood) (File S1). This gene encodes for a hormone involved in vascular homeostasis. Other clusters of genes upregulated in blood are involved in inflammatory and immune response process such as pentraxin-related gene (*PTX3;* 17.9 fold), cytokine-cytokine interaction, chemokine (C-C motif) receptor 3 (*CCR3;* 19.8 fold), interleukin 8 receptor beta (*IL8RB;* 14.5 fold) and interleukin 18 receptor 1 (*IL18R1;* 6.2 fold). Moreover, transcripts for B-lymphocyte antigen CD20 (*MS4A1*; 16.8 fold) and B-cell receptor CD22 precursor (*CD22*; 5.9 fold) were upregulated in the blood. There was also upregulation of genes involved in signal transduction including, among others, mitogen-activated protein kinase kinase kinase kinase (*MAP4K2*; 5.9 fold) and microtubule-associated protein-1S (*MAP1S*; 6.3 fold).

To address the question of whether CD14^+^ endometrial cells express genes that are characteristic of bovine macrophages, we compared the transcriptome of endometrial and blood CD14^+^ cells to the list of genes identified by Jensen et al. [22] as being expressed by bovine macrophages (Figure 2C). Of the 56 immune genes reported in that paper to be expressed in bovine macrophages, 52 were expressed by endometrial and blood CD14^+^ cells. Of these 52 genes, 5 were differentially expressed. Two genes were upregulated in endometrium [CD63 antigen (*CD63*) and *CCL8*] and three were upregulated in blood [*CD80*; *STAT1*; apoptosis (APO1) antigen 1 (FAS), member 6 (*TNFRSF6*)].

### Gene Ontology Analysis of Differentially Expressed Genes

The FatiGo web tool was used to perform a functional enrichment analysis to determine ontologies in which differentially regulated genes were more frequent in either endometrium or blood (Table S1). A total of 10 ontology classes were significant. In 4 cases, there was over-representation of differentially-expressed genes in endometrium (peptidase activity, endopeptidase activity, proteolysis, and extracellular region part) while in 6 cases there was over-representation of differentially-expressed genes in blood (zinc ion binding, phosphotransferase activity - alcohol group as acceptor, biopolymer metabolic process, signal transduction, nucleus, and cell communication).

### Differentially Expressed Pathways

Pathway Express software identified 33 pathways that were preferentially represented in the group of differentially expressed genes (Table S2). Among these were 6 pathways from the immune system group. Two pathways (complement and coagulation cascades, hematopoietic cell lineage) contained more upregulated genes in the endometrium than in the blood whereas three (toll-like receptor signaling, Fc epsilon RI signaling, and B cell receptor signaling) contained more upregulated genes in the blood. The pathway with the greatest impact factor, leukocyte transendothelial migration, was represented by 6 genes upregulated in endometrium and 6 upregulated in blood.

### Differentially Expressed Genes Characteristically Regulated During Macrophage Differentiation

**Figure 3:**
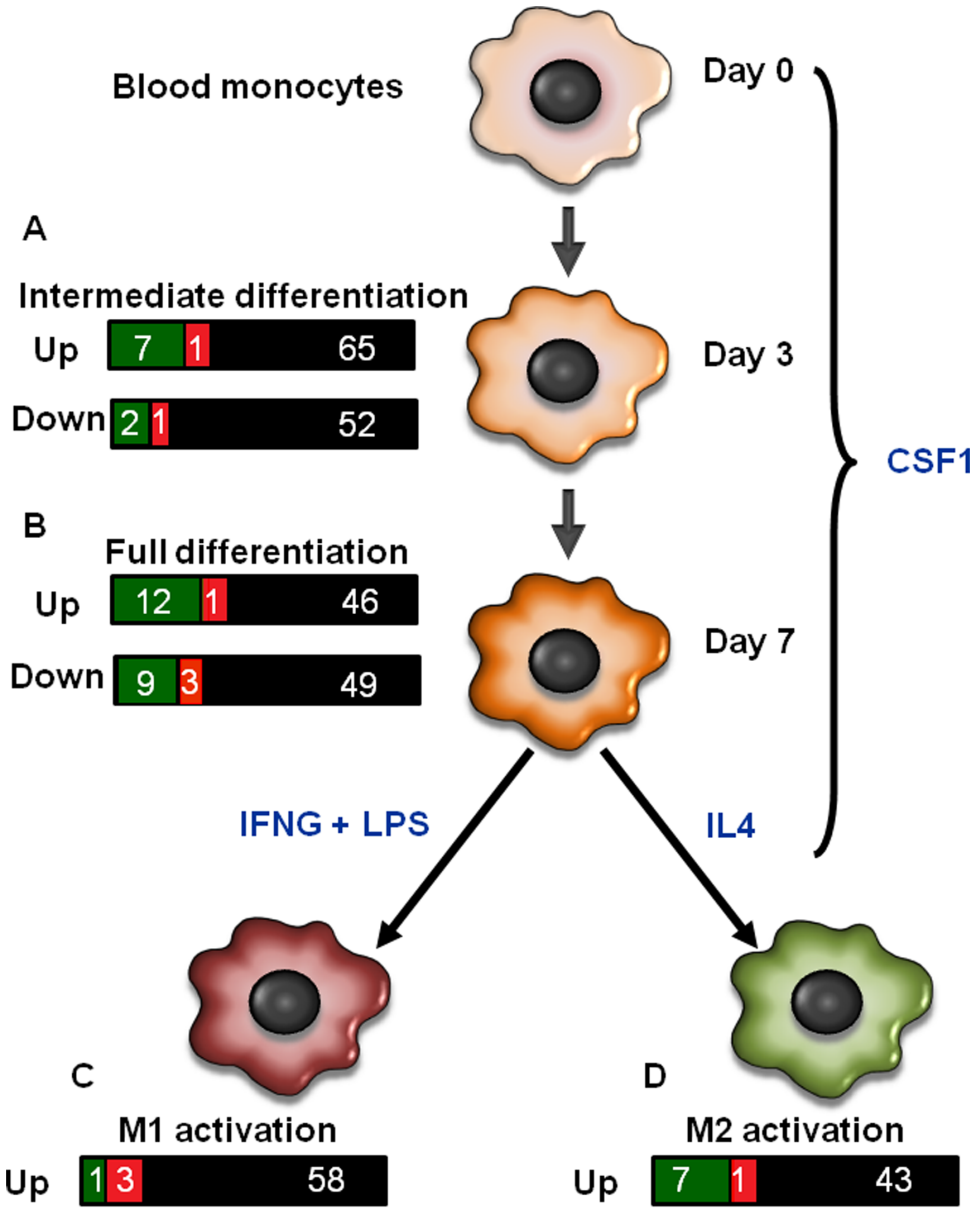
Graphical representation of differentially regulated genes in endometrial and blood CD14^+^ cells that have been reported to be differentially regulated during macrophage differentiation. Martinez et al. (23) identified genes that are differentially expressed in monocytes after 3 (intermediate differentiation; A) or 7 days (full differentiation; B) of culture with CSF1 to cause differentiation into macrophages. Afterwards, macrophages were polarized towards the classical activation (M1) pathway [C, achieved by culture with interferon-γ (IFNG) and lipopolyssacharide (LPS)) or alternative activation (M2) pathway (D, achieved by culture with interleukin 4 (IL4)). For each stage of differentiation, bars represent the number of genes in the Martinez et al. (23) data set that were found in the present data set of endometrial and blood CD14^+^ cells. Genes that are upregulated in endometrium are in green, genes upregulated in blood are in red, and genes that were not significantly upregulated are in black.

To determine the maturational state of endometrial macrophages as compared to blood monocytes, we evaluated which genes that are regulated during macrophage differentiation are differentially regulated between endometrial and blood CD14^+^ cells. The data set used as reference was developed by Martinez et al. [23]. In that study, human blood monocytes were cultured with CSF1 for 3 d (intermediate differentiation) or 7 d (full differentiation) to drive differentiation of monocytes to macrophages. Subsequently, CSF1 treated cells were cultured with either IFNG and lipopolysaccharide to cause differentiation to the classically-activated or M1 phenotype or IL4 to cause differentiation to the alternatively-activated or M2 phenotype (Figure 3).

The list of differentially-regulated genes in the current data set for which orthologs exist in the data set of Martinez et al. [23] are listed in Table S3 and a schematic representation of numbers of differentially-expressed genes identified by Martinez et al. [23] that were also upregulated in endometrium and blood is in Figure 3. Of 128 genes that were differentially expressed in monocytes after intermediate differentiation [23] 11 were differentially expressed between endometrium and blood (9 overexpressed in endometrium and 2 in blood). Of 120 genes differentially regulated after full differentiation [23], expression of 21 genes were higher in the endometrium and 4 were higher in the blood. Of 62 genes upregulated in M1 macrophages [23], 1 gene was upregulated in the endometrium and 3 were upregulated in the blood. Of 51 genes upregulated in M2 macrophages, 7 genes were upregulated in the endometrium and 1 gene was upregulated in the blood.

### Comparison to Genes Expressed by Human Decidual Macrophages

In the human, decidual macrophages possess a unique pattern of gene expression as compared to monocytes [21]. We asked if the genes that are upregulated or downregulated in human decidual macrophages are also differentially regulated in bovine endometrial macrophages. A total of 90% (107 of 120) of the genes found to be differentially-expressed in human decidual macrophages were present in the current data set. Of the 80 genes identified to be upregulated in decidual macrophages [21], 19 genes were significantly higher in the endometrium and 2 were higher in the blood. Of the 40 upregulated in blood monocytes [21], 3 genes were significantly higher in the blood and none were higher in the endometrium. The list of these genes is in Table 1.

**Table 1:** Differentially expressed genes in endometrial and blood CD14^+^ cells that are also differentially expressed in human decidual macrophages vs human blood monocytes.^a^

Description	Accession	Intensity		Fold change	P	Tissue^b^
		Endometrium	Blood			
Upregulated genes in decidual macrophages						
Granzyme A (*GZMA*)	NM_001001142	33196	276	120	0.00	Endo
Solute carrier organic anion transporter family, member 2B1 (*SLCO2B1*)	NM_174843	8667	173	50	0.00	Endo
Ribonuclease, RNase A family, 1 (*RNASE1*)	NM_001014386	5671	124	46	0.01	Endo
Secreted phosphoprotein 1 (*SPP1*)	NM_174187	4939	154	32	0.00	Endo
Collagen, type III, alpha 1 (*COL3A1*)	XM_588040	430	15	29	0.02	Endo
Mannose receptor, C type 1 (*MRC1*)	NM_002438	2246	93	24	0.00	Endo
Protein S (alpha) (*PROS1*)	NM_174438	1670	72	23	0.00	Endo
V-set and immunoglobulin domain containing 4 (*VSIG4*)	XM_865690	590	29	20	0.01	Endo
Complement component 1, q subcomponent, alpha polypeptide (*C1QA*)	NM_001014945	15126	793	19	0.00	Endo
Apolipoprotein E (*APOE*)	NM_173991	3576	208	17	0.00	Endo
Leprecan-like 1 (*LEPREL1*)	XM_589206	1776	125	14	0.03	Endo
Complement component 1, q subcomponent, beta polypeptide (*C1QB*)	XM_869694	29681	2122	14	0.00	Endo
Collagen, type I, alpha 2 (*COL1A2*)	NM_174520	892	72	12	0.04	Endo
Chemokine (C-C motif) ligand 8 (*CCL8*)	NM_174007	9546	1735	6	0.03	Endo
Chemokine (C-C motif) ligand 2 (*CCL2*)	NM_174006	18786	3526	5	0.01	Endo
NKG2C protein (*NKG2C*)	NM_001098163	665	139	5	0.03	Endo
Nerve growth factor receptor (TNFRSF16) associated protein 1 (*NGFRAP1*)	NM_014380	6512	1418	5	0.03	Endo
Endothelial PAS domain protein 1 (*EPAS1*)	NM_174725	7981	2114	4	0.05	Endo
Transmembrane protein 97 (*TMEM97*)	NM_001034403	6653	3454	2	0.05	Endo
Stathmin 1/oncoprotein 18 (*STMN1*)	XM_589478	384	2113	6	0.05	Blood
Nicotinamide riboside kinase 1 (*LOC510456*)	XM_587597	166	422	3	0.05	Blood
Upregulated genes in blood monocytes						
Selenoprotein X, 1 (*SEPX1*)	NM_001034810	7361	19546	3	0.03	Blood
Dipeptidase 2 (*DPEP2*)	XM_586714	140	328	2	0.05	Blood
Benzodiazapine receptor (peripheral) (*BZRP*)	NM_175776	2367	5508	2	0.04	Blood

a The list of genes that are differentially regulated in human tissues are obtained from reference 21.

b Tissue with higher expression in the current experiment. Endo signifies that expression was higher for endometrial CD14^+^ cells whereas Blood signifies higher expression in blood CD14^+^ cells.

### Quantitative RT-PCR

To validate the findings of the microarray analysis, qPCR was performed for 11 differentially expressed genes and a housekeeping gene (*RPL19* – bovine ribosomal protein L19). Analyses were performed on two sets of samples of mRNA. The first set was composed of mRNA from two pairs of endometrial and blood CD14^+^ cells that were used in the microarray because there was not mRNA enough to perform the qPCR analysis in all four pairs. The second set was composed of mRNA from an additional three pairs of endometrial and blood CD14^+^ cells that were not used for the microarray analysis.

In the first analysis (Table 2), with samples used in the microarray, 7 of 11 genes were significantly affected by tissue type [*PAG10; GZMA; HMOX1; MRC1; RNASE6; TSGA14; FCGR3A*]. In each case, expression was higher in endometrium, in agreement with results of the microarray analysis. In addition, an additional gene, SLCO2B1, tended to be higher in the endometrium (7.2-fold; P = 0.09). This gene was also more highly expressed in endometrium in the microarray analysis. Amounts of mRNA as determined by qPCR were not affected by tissue type for two other genes (*PTX3* and *MCP1*). In both cases, the fold-change was numerically higher for endometrium whereas, in the microarray analysis, expression was higher in blood.

**Table 2:** qPCR validation of 11 genes that were differentially regulated in the microarray analysis.

		RNA samples submitted to microarray and qPCR analysis							RNA samples submitted only to qPCR analysis						
		ΔCt^a^							ΔCt^a^						
Gene	Accession	Endometrium	Blood	Ste	ΔΔCt^b^	Foldchange	P	MC^c^	Endometrium	Blood	Ste	ΔΔCt^b^	Foldchange	P	MC^c^
*PAG10*	NM_176621	8.2	18.7	0.1	−10.4	1389	0.0003	Confirmed	7.5	19.1	1.2	−11.5	2964.0	0.002	Confirmed
*GZMA*	NM_001001142	6.8	16.5	0.9	−9.8	867	0.0112	Confirmed	7.7	16.1	1.5	−8.3	322.5	0.016	Confirmed
*HMOX1*	NM_001014912	12.4	16.8	0.1	−4.4	22	0.0002	Confirmed	11.9	16.0	1.3	−4.1	17.5	0.000	Confirmed
*MRC1*	XM_001252128	15.7	18.4	0.5	−2.7	6.7	0.0009	Confirmed	15.7	18.6	0.6	−3.0	7.8	0.065	Same direction
*TSGA14*	XM_583431	13.0	14.5	0.1	−1.5	2.9	0.0064	Confirmed	13.1	14.2	1.1	−1.1	2.1	0.523	Confirmed
*CD16a*	NM_001077402	10.1	11.0	0.2	−0.9	1.9	0.0115	Confirmed	11.4	11.1	1.4	0.3	1.2	0.889	Opposite direction
*CD163*	XM_613380	12.4	13.1	0.2	−0.7	1.6	0.0115	Confirmed	12.1	12.0	1.4	0.2	1.1	0.938	Opposite direction
*PTX3*	XM_614752	16.4	16.9	0.2	−0.5	1.4	0.0156	Opposite direction	17.4	16.4	0.6	1.0	2.0	0.351	Same direction
*MCP1*	NM_174006	15.2	15.3	0.1	0.0	1.0	0.0002	Opposite direction	15.4	14.5	2.3	0.8	1.8	0.814	Same direction
*RNASE6*	NM_174594	14.5	16.1	0.5	−1.6	3.0	0.1567	Same direction	11.1	14.2	1.0	−3.2	9.0	0.079	Same direction
*SLCO2B1*	NM_174843	15.8	18.7	0.1	−2.8	7.2	0.0887	Same direction	14.1	19.1	3.4	−5.0	31.3	0.365	Same direction

a ΔCt was calculated by the subtraction of the Ct value for the target gene from the Ct value for the housekeeping gene.

b ΔΔCt was calculated by the subtraction of the ΔCt value for the endometrium from the ΔCt value for the blood.

c MC  =  Microarray confirmation.

For the group of independent samples (Table 2), 4 genes were significantly upregulated in endometrium compared with the blood (*PAG10; GZMA*, *TSGA14 and HMOX1*) and all of these genes were higher in the endometrium in the microarray analysis. An additional 5 genes show differences in the same direction as for the microarray although differences were not significant (*MCP1*, *RNASE6*, *SLCO2B1*, *PTX3 and MCP1*). Two genes were not significantly affected by tissue and differences were in the opposite direction as for the microarray analysis (*CD163*, *CD16*).

## Discussion

That accumulation of macrophages in the endometrium is widespread phylogenetically and of large magnitude [1]–[8] is evidence that these immune cells play an important role in supporting the growth and survival of the conceptus. Using global transcriptomal analysis, macrophages in the interplacentomal endometrium of the cow exhibit patterns of gene expression that suggest important roles in immune regulation, tissue remodeling, angiogenesis, and apoptosis. Moreover, these functional properties are likely to be a general feature of endometrial macrophages and not a specific role of these cells in the cow because the pattern of genes that are differentially regulated in endometrial macrophages of the cow is similar to genes that are differentially regulated in decidual macrophages in the human [21].

To assess gene expression, cells expressing CD14 were purified from endometrium and blood. CD14 is a co-receptor for bacterial lipopolysaccharide and is expressed on monocytes, macrophages and neutrophils [24], [25]. Previously [7], [8], CD14^+^ cells in endometrium of pregnant cows were identified as macrophages because they also express CD68, a lysosomal-associated protein that is expressed on monocytes, macrophages and dendritic cells [26]. In the present study, endometrial CD14^+^ cells had a pattern of gene expression that was largely similar to that of blood CD14^+^ cells (Figure 1A). Moreover, given the similarity in gene expression between endometrial and blood CD14^+^ cells, it is likely that the massive infiltration of CD14^+^ cells in the pregnant endometrium is a result of the recruitment from blood monocytes. Interestingly, the proportion of peripheral blood mononuclear cells that are monocytes decreases in late pregnancy in the cow [7], possibly as a result of recruitment to the endometrium.

The pattern of differential gene expression is indicative of changes in cell migratory function as monocytes move from blood to endometrial stroma. Blood monocytes appear to have a high capacity for responsiveness to external signals that ensures activation of migration into tissues upon chemotactic signals from tissues. There was a preponderance of upregulated genes in blood for several differentially-regulated pathways involved in cell signaling including toll-like receptor signaling, Fc epsilon RI signaling, B cell receptor signaling, phosphatidylinositol signaling, JAK-STAT signaling, MAPK signaling, wnt and cytokine-cytokine receptor signaling and ErbB signaling (Table S2). Among the upregulated genes that would promote activation of migration into tissues are various cytokine receptors (Table S2). One pertinent gene is integrin alpha 4 (*ITGA4*), that encodes for the alpha 4 subunit of the VLA-4 receptor and which promotes transendothelial migration by interacting with endothelial cell surface receptors VCAM-1 and JAM2 [27]. Another is the gene for the nuclear repressor protein glucocorticoid receptor DNA binding factor-1 (*GRLF1*) that antagonizes the inhibitory effect of TGFB1 on monocyte migration [28]. Several genes involved in cell motility were also upregulated in blood including several myosin genes (*MYL2*, *MYLPF*, and *MYH10*) and two regulators of the actin cytoskeleton, diaphanous homolog 1 (*DIAPH1*) and paxillin (*PXN*).

Once macrophages are in the endometrium, the above-mentioned genes involved in cell signaling and diapedesis become downregulated. Other genes involved in motility increase, including thymosin beta 4 (*TMSB4X*), which promotes cell motility through sequestration of G-actin monomers during actin-cytoskeletal organization [29] and enabled homolog (*ENAH*), which blocks capping of actin filaments and promotes filopodia formation [30]. These changes may indicate a different pattern of cell motility once monocytes enter the endometrium. At the same time, endometrial CD14^+^ cells gain increased capacity for interacting with other cellular and extracellular components of the endometrium as indicated by increased expression of genes encoding for cell adhesion molecules and ECM-receptor interaction pathways (Table S2). Cell adhesion proteins on endometrial macrophages likely function to facilitate regulation of other immune cells. For example, E-cadherin (*CDH1*), a gene upregulated in endometrium, promotes macrophage – T cell interactions [31].

Endometrial CD14^+^ cells may themselves promote migration of monocytes and other leukocytes into the endometrium as indicated by upregulation of *CXCL14* and chemokines *CCL8*, *CCL22* and *CCL24*. The platelet derived growth factor B polypeptide (*PDGFB*) was also upregulated in endometrium. PDGF directs movement of neutrophils and macrophages [32].

Despite the identification of CD14^+^ cells as macrophages, some of the upregulated genes in endometrium were placental and uterine genes such as pregnancy associated glycoproteins [33], prolactin related proteins [34] and uterine serpin [35]. Endometrial CD14^+^ cells were 90–95% pure, and the presence of placental or endometrial genes could reflect contamination of the CD14^+^ population with trophoblast, endometrial epithelium or endometrial stroma. In species where endometrium undergoes a decidual response, decidualized stroma express cytokines and chemokines characteristically secreted by immune cells [36], [37]. Perhaps similar differentiation pathways exist in species like cattle so that CD14 becomes upregulated in non-myeloid cells.

Once migrating into the endometrium, at least a subset of CD14^+^ monocytes undergo differentiation. This is so because many of the genes identified by Martinez et al. [23] as being upregulated in macrophages during differentiation were upregulated in endometrial CD14^+^ cells as compared to CD14^+^ cells in blood. We hypothesized that, in the absence of infection, endometrial macrophages would be preferentially activated along the M2 activation pathway so that that differentiation results in macrophages that promote inhibition of inflammation, angiogenesis, and tissue remodeling [38]–[40]. Indeed, as detailed in the next paragraphs, there is evidence for bias of endometrial macrophages towards M2 activation. Nonetheless, it is likely that there is more than one subpopulation of endometrial macrophage resident in the endometrium of pregnant cows. Some subpopulations of endometrial macrophages may undergo a differentiation process that is unique for that tissue.

Seven of 51 genes upregulated in M2 activated macrophages [23] were upregulated in endometrial CD14^+^ cells. One of these genes was glycine amidinotransferase (*GATM*) which competes with the inducible form of nitric oxide synthase for arginine and thereby reduces production of the proinflammatory molecule nitric oxide [41]. Two C-type lectins characteristically activated in M2 macrophages, mannose receptor C1 (*MRC1*) and C-type lectin domain family 7, member A (*CLEC7A* or dectin-1) were also upregulated in endometrial CD14^+^ cells. Activation of MRC1 can lead to inhibition of secretion of IL12 (stimulates NK cell and T-cells) and increased secretion of the anti-inflammatory cytokines IL8, IL10, IL1 receptor antagonist and IL1 receptor type II [42]. In contrast, CLEC7A, which recognizes fungal carbohydrates, can participate in release of inflammatory cytokines [43]. Another gene upregulated in endometrium was prostaglandin G/H synthase (*PTGS1* or cyclooxygenase 1) which is a key enzyme in prostaglandin, prostacyclin, and thromboxane A2 biosynthesis. These eicasonoids have been implicated in immunosuppression (prostaglandin E2) and angiogenesis (prostaglandins E2, I2, F_2α_, and thromboxane A2) [44].

The one upregulated gene in endometrium that could be classified as characteristic of M1 macrophages (complement factor B, *CFB*) also encodes for a protein that can have anti-inflammatory properties. Cleavage of CFB by complement factor D liberates the Ba fragment that inhibits proliferation of B cells [45].

In addition to the genes identified by Martinez et al. [23], other genes expressed in M2 macrophages were upregulated in endometrium including *CD163*, which is a scavenger receptor that, in its soluble form, exerts anti-inflammatory actions [46] as well as the anti-inflammatory cytokines *CCL22* and *CCL24* that are produced by M2a macrophages [39]. *CCL2*, an M1 cytokine [39], was also upregulated in endometrium. Another gene upregulated in M2 macrophages, E-cadherin (*CDH1*), was one of the top 100 upregulated genes in the endometrium. This cell adhesion protein promotes macrophage fusogenic activity and macrophage – T cell interactions [31]. Two other genes with anti-inflammatory activity were upregulated in endometrium. TMSB4 can be oxidized by macrophages exposed to glucocorticoteroids to form thymosin β4 sulfoxide that has anti-inflammatory properties [47]. Fibroblast growth factor 1 (*FGF1*), another gene upregulated in endometrium, inhibits expression of monocyte adhesion molecules on endothelial cells involved in transendothelial movement and may be anti-inflammatory [48].

One function of M2 macrophages is to promote tissue remodeling [40] and analysis of the transcriptome of endometrial vs blood CD14^+^ cells is consistent with endometrial CD14^+^ cells being actively involved in this process. There was over-representation of differentially-expressed genes in endometrium for three ontologies related to proteolysis (peptidase activity, endopeptidase activity, and proteolysis).

The upregulation of granzymes (*gzma*, *GZMA* and *GZMB*) in endometrial CD14^+^ cells is consistent with a role for these cells in induction of apoptosis. Apoptotic cells are common in the placenta, especially at the edges of the placentomes and in the interplacentomal region [49]. While the bovine trophoblast is considered to be non-invasive and not capable of crossing the basement membrane of the luminal epithelium of the endometrium, it is possible that endometrial macrophages remove placental cells that stray past this barrier. Apoptosis could also be a signal for regulation of macrophage function. Abrahams *et al.* [50] hypothesized that ingestion of apoptotic trophoblast cells causes decidual macrophages to develop an immunosuppressive phenotype. Another possible interpretation of the upregulation of granzymes is that a portion of the CD14^+^ cells in endometrium are natural killer cells since decidual natural killer cells in human contain granzyme A [51].

Validation of microarray results with qPCR validation resulted in differential expression being confirmed for 7 of 11 genes evaluated. Examination of differences between endometrial and blood CD14^+^ cells using qPCR of an independent data set indicated a smaller degree of agreement with microarray results (4 of 11 genes) but a total of 9 of 11 genes showed differences in the same direction as the results from microarray hybridization. The independent samples were from a slightly earlier stage of pregnancy and this fact could have resulted in some discrepancies with the microarray data set.

In summary, endometrial CD14^+^ cells express genes characteristically found in macrophages. Moreover, analysis of the transcriptome of these cells indicates that at least a subpopulation of the cells differentiates along an M2 activation pathway and that the cells play roles in immune regulation, tissue remodeling, angiogenesis, and apoptosis. These roles are likely of broad relevance to mammalian reproduction rather than to be specific properties of pregnancy in the cow. Endometrial macrophages in the human also undergo M2 activation [3], [21]. In addition, 20% of the genes found by Gustafsson et al. [21] to be differentially expressed between decidual and blood macrophages were also differentially expressed in the same direction in cow. Thus, despite substantial evolutionary divergence in reproductive patterns, the properties of the endometrial macrophage during pregnancy have been at least partially conserved.

## Materials and Methods

### Materials

Mouse anti-bovine CD14 (clone MM61A, clarified ascites fluid, 10 µg/ml) was obtained from VMRD, Inc. (Pullman, WA, USA). The Ab was tagged with labeled Fab fragments against mouse IgG conjugated to Alexa Fluor 488 using the Zenon® Mouse Labeling IgG kit from Invitrogen Molecular Probes (Eugene, OR, USA) as per manufacturer's instructions.

Fico/Lite LymphoH was purchased from Atlanta Biologicals (Norcross, GA, USA). Fetal bovine serum was purchased from Sigma-Aldrich (St. Louis, MO, USA) or Pel-Freez Biologicals (Rogers, AR, USA). Tissue Culture Medium-199 (TCM-199), bovine serum albumin (BSA) Fraction-V, Dulbecco's PBS (DPBS), and collagenase type I was purchased from Sigma-Aldrich (St. Louis, MO, USA). Other reagents were from Sigma-Aldrich or Fisher Scientific (Pittsburgh, PA, USA).

### Collection of Endometrium and Blood

For microarray analysis, the uterus and cardiac blood were obtained at the time of euthanasia at a local abattoir from four cows at an estimated 164, 239, 242 and 248 days of pregnancy, respectively (i.e., dating based on fetal crown-rump length [52]). Blood was collected into heparinized tubes. Blood and the uterus were transported to the lab on ice within 1.5 h after slaughter. The uterus and cardiac blood from three additional cows at an estimated 156, 169 and 176 days of pregnancy were collected as described above for RNA collection to be used for quantitative real time PCR (qPCR) analysis.

### Isolation of Mononuclear Leukocytes in Blood

PBMCs were obtained from blood as follows. Blood (10 ml) was centrifuged at 600 g for 30 min to obtain the buffy coat. This layer was mixed with 2 ml TCM-199 and the cell suspension was transferred to the top of 2 ml Fico/Lite LymphoH placed in a 15 ml conical tube. Cells were centrifuged at 600 g for 30 min. Mononuclear cells were collected at the interface of the Fico/Lite LymphoH and media, centrifuged at 600 g for 10 min, resuspended in DPBS, and evaluated with a hemacytometer to determine cell concentration and viability (trypan blue exclusion). Cells were resuspended to a final concentration of 5×10^7^/ml in staining buffer (DPBS supplemented with 0.1% (w/v) BSA).

### Preparation of Cell Suspension from Endometrium

A suspension of cells was prepared from interplacentomal endometrium dissected from the uterine horn containing the fetus. Dissected endometrium was finely minced in DPBS using a sterile scalpel blade and a petri dish and then placed into a 50 ml sterile culture tube containing 25 ml digestion medium (TCM-199 supplemented with 150 U/ml type I collagenase). Tissue was incubated at 37°C under gentle rotation for 15 min. Undigested tissue was allowed to settle for 5 min; the supernatant fraction was then collected by aspiration, and 25 ml of fresh digestion medium was added to the tissue fraction. The remaining tissue was incubated for an additional 15 min under rotation, and the supernatant fraction produced after settling was pooled with the previous supernatant fraction. Cells in suspension in the supernatant fraction were collected by filtration through a sterile 100 µm cell strainer into 50 ml sterile culture tubes. Cells were centrifuged at 110× g for 5 min, resuspended in 5 ml TCM-199 supplemented with 10% (v/v) fetal bovine serum, analyzed for cell number using a hemacytometer, and diluted to a final concentration of 5×10^5^/ml in TCM-199. A 10 ml aliquot of the dispersed endometrial cell suspension was layered onto 5 ml Fico/Lite LymphoH in a 15 ml conical tube. Cells were centrifuged at 600 g for 30 min. The interface containing mononuclear cells was collected and resuspended in 10 ml of staining buffer, washed once, resuspended in staining buffer, and evaluated for cell concentration and viability (trypan blue exclusion) using a hemacytometer. Cells were then resuspended to a final concentration of 5×10^6^/ml.

### Purification of CD14^+^ Cells

Endometrial cells and PBMC were separately subjected to flow cytometry and cell sorting to prepare CD14^+^ cells. The entire pool of cells obtained as described above was placed into a 15 ml conical tube containing staining buffer, washed twice with 5 ml staining buffer, and resuspended in the smallest volume possible with staining buffer. Cells were then labeled by incubation with Zenon-labeled anti-CD14 by adding 0.1 µg labeled Ab per 5×10^6^ cells. A Zenon-labeled mouse IgG1 at the same concentration as the primary Ab was incubated with another aliquant of cells to determine nonspecific labeling. Cells and labeled Ab were incubated at room temperature for 30 min. After incubation, cells were diluted to 5 ml with staining buffer to 5×10^6^ cells/ml and subjected to cell sorting using flow cytometry with a FACSCAria Cell Sorter supported by DiVa software V.6.1.2 (BD Biosciences, San Jose, CA, USA). Sorting was performed with a 100 micron nozzle and pressure of 20 psi. Cells were gated on the basis of forward and side scatter to analyze the monocyte region. Two rounds of cell sorting were performed. The first round of sorting, at a high flow rate, was performed to maximize yield of CD14^+^ cells while the second step was designed to maximize purity of the CD14^+^ cells at low flow rate.

### RNA Purification

Sorted CD14^+^ cells were centrifuged at 600 g for 5 min and total cellular RNA was extracted using the using the RNeasy Micro kit (Qiagen Inc, Valencia, CA, USA). Concentration of total cellular RNA was determined using the Nanodrop 1000 spectrophotometer (Thermo Scientific, Waltham, MA) and integrity determined by Agilent 2100 Bioanalyzer with RNA 6000 Pico LabChip kit (Agilent Technologies, Santa Clara CA, USA). Only samples that showed high RNA integrity (RIN >7) were used for the microarray hybridization and quantitative PCR analysis. Extracted RNA was stored at −80°C until microarray analysis.

### Microarray Hybridization

Four pairs of samples of blood and endometrial CD14^+^ cells were subjected to transcriptional profiling using the Bovine Oligo Microarray Chip (Bovine 4X44K G2519F) from Agilent (Santa Clara CA, USA). Each pair was collected from the same animal. The array contains 43,803 bovine probes representing 20,767 unique 60-mer probes and approximately 19,500 distinct bovine genes arranged on a slide as 4 arrays in a 4X44K format. The probes were developed by clustering more than 450,000 mRNA and EST sequences of the bovine genome (btau 2.1). All microarray protocols were carried out by Mogene LLC (St. Louis, MO, USA), an Agilent Certified Service Provider.

Prior to microarray hybridization, 10–50 ng of total RNA were amplified using the WT-Ovation Pico RNA Amplification Kit (NuGen Technologies, Inc, San Carlos, CA, USA) according to the manufacturer's protocol. Two micrograms of amplified material was labeled with Cy3 or Cy5 using Agilent Genomic DNA Enzymatic Labeling Kit (Agilent Technologies, Santa Clara CA, USA) and 1.5 µg of each labeled sample were co-hybridized to the array for 17 h at 65°C at 10 rpm in a rotating oven in an ozone-free room using SureHyb chambers from Agilent (Agilent Technologies, Santa Clara CA, USA). Wash conditions were as outlined in the Agilent processing manual and the arrays were scanned using an Agilent G2505B scanner (Agilent Technologies, Santa Clara CA, USA). Two technical replicates were performed. For the first replicate, RNA samples from blood were labeled with Cy3 and RNA from endometrium was labeled with Cy5. For the second technical replication, 3 samples of RNA from blood were labeled with Cy5 and their matching pairs of RNA from endometrial were labeled with Cy3 while the fourth pair used Cy3 for blood and Cy5 for endometrium.

### Analysis of Microarray Data

The microarray image extraction and data pre-processing were performed using Agilent's Feature Extraction software v 9.5 (Agilent Technologies, Santa Clara CA, USA). The intensity of each spot was summarized as the median pixel intensity followed by log transformation. The lowess normalization was done within-array lowess on median of the intensities of the spots. The JMP® Genomics 3.1 for SAS® 9.1.3 software (SAS Inst., Inc., Cary, NC) was used. The quantile normalization method for data global normalization was performed. Differentially regulated genes were identified by least-squares ANOVA by the PROC ANOVA procedure of JMP® Genomics 3.1 for SAS® 9.1.3. The model included animal, replicate, and tissue source (blood or endometrium). Fixed effects included tissue and replicate and random effects included animal. Tissue x animal was the error term for tissue. Correction for false discovery rate was performed by the Benjamini and Hochberg method [53] with a maximum false discovery rate of 0.01. Genes with a minimum intensity of 16 were considered to be expressed. Only genes with at least 2-fold difference and a probability of P≤0.05 for a tissue difference were considered differentially expressed. Expression values were deposited in the Gene Expression Omnibus repository as Series number GSE20190 (www.ncbi.nlm.nih.gov/geo/).

### Gene Ontology Analysis

An analysis was performed using FatiGo (a web-based program for functional profiling; www.babelomics.org) to indentify functional enrichment by comparing the two lists of differentially regulated genes from endometrium and blood by means of a Fisher's exact test [54]. Furthermore, Pathway Express (http://vortex.cs.wayne.edu/ontoexpress) was used to identify relevant pathways represented by the differentially regulated gene set. Pathway Express calculates a probability (P) value, based on the density of genes that belong to a determined pathway using the Kyoto Encyclopedia of Gene and Genome database [55]. It also calculates a gamma P value using the perturbation factor for each input gene in the pathway that accounts for the normalized fold change of the gene and the number and the amount of perturbation of genes at downstream positions on the pathway. Both P- and gamma P-values are combined to generate the impact factor which was used to rank the pathways according to their biological significance. A pathway was considered to be significantly affected by tissue source if the gamma P-value ≤0.05.

### Quantitative Real-Time PCR

Quantitative PCR was performed on 11 genes found to be differentially expressed by microarray hybridization as well as a housekeeping gene used as an internal control (bovine ribosomal protein L19; *RPL19*). Samples from a total of five pairs were analyzed by qPCR. These included 2 pairs of samples that were also used for the microarray analysis and 3 pairs of new samples. We could not use samples from all 4 pairs used in the microarray analysis for the qPCR validation due to insufficient quantities of RNA.

Primers and probes are shown in Table 3. The qPCR analyses were carried out by Mogene LLC. The hydrolysis probe based system was designed using the online tool “PrimeTime qPCR Assays” by IDT DNA (www.idtdna.com) and the primers and probes were purchased from the same company. The final primer and probe concentration was 200 nM. All probes had a 5′ 6-Fam label and a 3′ IA-Black Quencher. The reaction was designed for a total reaction volume of 25.0 µl. The template cDNA (25 ng per reaction) was added to each reaction in 5.0 µl. The Applied Biosystems Taqman Gene Expression Mastermix (Foster City, CA, USA) was used. Cycling conditions were 95°C for 10 min, and 40 cycles at a 95°C melting temperature for 15 sec. and 60°C for 1 min. Each sample was assayed in 3 replicates.

**Table 3:** Primer/probe sets used for qRT-PCR.

Gene Name	Accession	Primer/Probe	Sequence (5′-3′)
CD163 molecule (*CD163*)	NM_001077402	forward^a^	AAT TTC GTG GAC AGA GTT CTC
		probe^b^	/56-FAM/AGA CAG CGG CTT GCA GTT TCC/3IABlk_FQ
		reverse^c^	AAT TTC GTG GAC AGA GTT CTC
Mannose receptor C1 (*MRC1*)	XM_001252128	forward^a^	TCG AGT TGA GCC ACT TCA
		probe^b^	/56-FAM/CCA CAC CCA CAT TCC TTC AAC ATT TCT/3IABlk_FQ/
		reverse^c^	TCT TTC ACC AGA GGG ATC AC
Granzyme A (*GZMA*)	NM_001001142	forward^a^	AAG ACG CTA CAT GGC TCT
		probe^b^	/56-FAM/CGC TCA TTG TGA CCT GAA GGG C/3IABlk_FQ/
		reverse^c^	ATG GGA TGT AGA GTG GGC
Heme oxygenase (decyclizing) 1 (*HMOX1*)	NM_001014912	forward^a^	CGG AGA ATG CAG AGT TCA
		probe^b^	/56-FAM/AAG GTT TTA AGC TGG TGA TGG CGT C/3IABlk_FQ/
		reverse^c^	TGT TGC GTT CGA TCT CCT
Chemokine (C-C motif) ligand 2; CCL2 (*MCP1*)	XM_614752	forward^a^	AGA GGC TGT GAT TTT CAA GAC
		probe^b^	/56-FAM/AGT TAT GTG CAG ACC CCA AGC AGA/3IABlk_FQ/
		reverse^c^	GGT TGT GGA GTG AGT GCT
Fc fragment of IgG, low affinity IIIa, receptor (*CD16A*)	NM_001077402	forward^a^	GAA TGG AGG GAT GGC AAA
		probe^b^	/56-FAM/TTA GGA CAA ATG GAG GCA TCT CTG GG/3IABlk_FQ/
		reverse^c^	ACA GAG TTG GGT GAA GGA TC
Ribonuclease k6 (*RNASE6*)	NM_174594	forward^a^	CCC CTT ATC ATT TGG TTC CTG
		probe^b^	/56-FAM/AGG TTG TTT AAA TCA CTT TGC TTC TCG CT/3IABlk_FQ/
		reverse^c^	AAA TCA ATA AAA GAC AAG AAA ATC AGA G
Testis specific, 14 (*TSGA14*)	XM_583431	forward^a^	AGA ATC CCC GCT CAC TG
		probe^b^	/56-FAM/CAA GGT CCT TTA CTT CCA GGG TTT GC/3IABlk_FQ/
		reverse^c^	GAA AAC ATT TAT TTG ACT AAG GCA G
Pregnancy-associated glycoprotein 10 (*PAG10*)	NM_176621	forward^a^	GCC CAA GCT TAC ATC CAA AG
		probe^b^	/56-FAM/TCT CCG ACT CGT TCA CAC GCT/3IABlk_FQ/
		reverse^c^	GAG AAA TAC AGC CTC AGG AAG
Solute carrier organic anion transporter family, member 2B1 (*SLCO2B1*)	NM_174843	forward^a^	ATC CAG AGT GTG AGC TGT
		probe^b^	/56-FAM/CCC AAT ACC CAA TTC CAG GCA AAG G/3IABlk_FQ/
		reverse^c^	CAA GTT AGC GGA AGA CTC AC
Ribosomal protein L19 (*RPL19*)	NM_001040516	forward^a^	CTG AAG GTG AAG GGT AAC G
		probe^b^	/56-FAM/AGG CAG ACA AGG CTC GCA AGA/3IABlk_FQ/
		reverse^c^	GGG CTT CCT TGG TCT TAG A

a Forward = sense (5′) primer.

b Each probe was synthesized with a 5′ 6-FAM reporter dye and 3′ IA-Black quencher.

c Reverse = antisense (3′) primer.

The level of expression of each tested gene was calculated using the ΔCt method with normalization to the RPL19 housekeeping gene. Treatment effects were analyzed by least squares analysis of variance using the General Linear Models procedure of SAS (SAS for Windows, version 9.3, SAS Institute Inc., Cary, NC, USA). The animal x tissue interaction term was used as the error to test effect of tissue. Results are presented as least-squares means ± standard errors.

## Supporting Information

Table S1 Ontologies where the incidence of frequency of overexpressed genes in the endometrium was different than the incidence of overexpressed genes in the blood.
(0.08 MB PDF)

Table S2 Pathways subject to differential regulation.
(0.11 MB PDF)

Table S3 Differentially regulated genes in endometrial and blood CD14+ cells that were also differentially regulated in the intermediately-differentiated, fully differentiated, M1 and M2 activated macrophage.
(0.09 MB PDF)

File S1 List of differentially expressed genes.
(0.16 MB XLSX)
