# Supplementary material for: Differentiation of the Endometrial Macrophage during Pregnancy in the Cow
Source: PLoS One. 2010 Oct 7;5(10):e13213. doi: 10.1371/journal.pone.0013213 (PMC2951363; doi:10.1371/journal.pone.0013213)
Supplement: Table S1 — Ontologies where the incidence of frequency of overexpressed genes in the endometrium was different than the incidence of overexpressed genes in the blood. (0.08 MB PDF) [file pone.0013213.s001.pdf]

Table S1. Ontologies where the incidence of frequency of overexpressed genes in the endometrium was different than the incidence of overexpressed genes in the blood.[a]

Ontology term	Genes differentially expressed in the endometrium (E) and blood (B)	Percentage of genes in the ontology		P
		Endometrium	Blood	
Molecular function				
Peptidase activity (GO:0008233)	E: *CTSK; CTS; DPP4; GZMA; PAG3; PAG4; PAG5; PAG6; PAG10; PAG11; PAG15; PAG17; PAG18; PAG1B; PAG21; PLAT; PSEN1; ST14*	16.1	0.0	0.003
	B: none			
Endopeptidase activity (GO:0004175)	E: *CTSB; CTSK; CTSL; CTSZ; DPP4; GZMA; GZMA; PAG10; PAG11; PAG15; PAG17; PAG18; PAG1B; PAG21; PAG3; PAG4; PAG5; PAG6; PAG9; PLAT; ST14*	15.3	0.0	0.016
	B: none			
Zinc ion binding (GO:0008270)	E: *FBLIM1;egr1;RNF128;NT5E ;NR1H3*	5.6	26.9	0.048
	B: *CA4; FGD6; MYNN; NR1H2; PCGF1; PCGF4; PHF17; PPARD; PRKCB1; RABEX5; RARA; SEC23B; ZFX; ZNF410*			
Phosphotransferase activity; alcohol group as acceptor (GO:0016773)	E: *IHPK3; STK25*	1.5	13.2	0.052
	B*: CSNK1A1; CSNK1E; DGKH; FYN; IPMK; LOC538702; PDIK1L; PRKCB1; RIOK3*			
Biological process				
proteolysis (GO:0006508)	E: *C1QA; CTSB; CTSK; CTSL; CTSZ; DPP4; gzmA; GZMA ; PAG10; PAG11; PAG15; PAG17; PAG18; PAG1B; PAG21; PAG3; PAG4; PAG5; PAG6; PAG9; PLAT; PSEN1; RNF128; ST14;*	25.0	0.0	0.001
	B: none			
signal transduction (GO:0007165)	E: *ANG; EDG2; EDNRB; EPAS1; FZD3; GRIM19; ITGB6; OXTR; PGDH; PRLR; PSCD4; PSEN1; RAB13; RRAS;*	11.3	34.3	0.013
	B: *ARNT; ASB6; CDK5R1; CNIH4; CSNK1A1; CSNK1E; DGKH; FGD6; ITGA4; MS4A1; OPN1SW; PPP2CA; PRKCB1; RAB11A; RAB3A; RABIF; RASGRP4; STAT3; STAT5A; STAT5B; TAC3; TIMAP; TNFRSF1B; TNFRSF6; UPK1A;*			
biopolymer metabolic process (GO:0043283)	E: *ANG; AR; CBX6; CTGF; EGR1; ELF5; EPAS1; ERCC5; FOS; FOXA3; GGCX; GRIM19; HAND1; JUN; MSX1; NR1H3; PSEN1; RNF128; ST3GALIV; STK25; TFB1M;*	16.9	41.1	0.013
	B: *ARNT;B3GNT5; BAT3; CBX7; CSNK1A1; CSNK1E; DUSP12; IRF1; L41691;MYB; MYNN; NR1H2; OPN1SW; PDIK1L; PHF17; POP4; PPARD; PPIL1; PPP2CA; PRKCB1; RARA; RPP38; SNURF; STAT3; STAT5A; STAT5B; TREX1; UBE2B; UFM1; ZFX;*			

cell communication (GO:0007154)	E: *ANG; APOE; EDG2; EDNRB; EPAS1; FZD3; GJB1; GRIM19; ITGB6; OXTR; PGDH; PRLR; PSCD4; PSEN1; RAB13; RRAS*;<br>B: *ARNT; ASB6; CDK5R1; CNIH4; CSNK1A1; CSNK1E; DGKH; FGD6; ITGA4; MS4A1; OPN1SW; PPP2CA; PRKCB1; RAB11A; RAB3A; RABIF; RASGRP4; STAT3; STAT5A; STAT5B; TAC3; TIMAP; TNFRSF1B; TNFRSF6; UPK1A;*	12.7	33.3	0.042
Cellular component				
nucleus (GO:0005634)	E: *ANG; AR; BANF1; CBX6; egr1; ELF5; EPAS1; ERCC5; FOS; FOXA3; GRIM19; HAND1; JUN; MSX1; NR1H3; PSEN1; UACA;*<br>B: *ARNT; CBX7; CCNE2; CDK5R1; IRF1; L41691; MYB; MYNN; NR1H2; PHF17; POLR2J2; POP4; PPARD; PPIL1; PPP2CA; RARA; RBM15B; RPP38; SNURF; STAT3; STAT5A; STAT5B; TAF11; TREX1; UBE2B; UFM1; ZFX;*	29.82	64.29	0.019
extracellular region part (GO:0044421)	E: *ANG; APOE; CCL2; CCL8; COL1A2; CTGF; DCN; GJB1; MAGP2; SPP1;*<br>B: none	8.85	0.00	0.079

[a]Analysis was performed using FatiGo—a web tool for finding significant associations of Gene Ontology terms with groups of genes.
