# Supplementary material for: Differentiation of the Endometrial Macrophage during Pregnancy in the Cow
Source: PLoS One. 2010 Oct 7;5(10):e13213. doi: 10.1371/journal.pone.0013213 (PMC2951363; doi:10.1371/journal.pone.0013213)
Supplement: Table S2 — Pathways subject to differential regulation. (0.11 MB PDF) [file pone.0013213.s002.pdf]

**Table S2.** Pathways subject to differential regulation.[a]

| Pathway Name | IF[b] | Genes overexpressed in endometrium (E) and blood (B) | IG/GP[b] | P[b] | γP[b] |
|---|---|---|---|---|---|
| **Immune system** | | | | | |
| Leukocyte transendothelial migration | 895 | E: *PXN; VCAM1; THY1; CLDN3; CLDN1; CLDN8* | 12/119 | 0.02 | 0.00 |
| | | B: *GRLF1; MYL2; ITGA4; PIK3R5; PIK3R3; MYLPF* | | | |
| Toll-like receptor signaling pathway | 11 | E: *MAPK3; FOS; JUN; TLR3; SPP1* | 16/102 | 0.00 | 0.00 |
| | | B: *MAP2K4; RELA; MAP2K3; PIK3R3; STAT2; TIRAP; MAPK3K8; IFNAR1; TRAF3; CD80; PIK3R5* | | | |
| Complement and coagulation cascades | 11 | E: *C1QA; C1QB; C1QC; C3AR1; CD59; CFB; PLAT; PROS1; SERPINA5; TFPI; FGB* | 11/69 | 0.00 | 0.00 |
| | | B: none | | | |
| Hematopoietic cell lineage | 5 | E*: CD59; TFRC; ITGA6; CD7; CSFR1; MME* | 9/87 | 0.03 | 0.03 |
| | | B: *MS4A1; CD22; ITGA4* | | | |
| Fc epsilon RI signaling pathway | 5 | E: *MAPK3; RAC3; PLA2G3* | 9/78 | 0.01 | 0.04 |
| | | B: *PIK3R5; PIK3R3; GRB2; MAP2K3; MAP2K4; FYN* | | | |
| B cell receptor signaling pathway | 5 | E*: RAC3; JUN; FOS* | 8/65 | 0.04 | 0.04 |
| | | B: *CD79A; RELA; PIK3R5; PIK3R3; CD22* | | | |
| **Signaling molecules and interaction** | | | | | |
| Cell adhesion molecules (CAMs) | 632 | E: *ALCAM; SIGLEC1; ITGA6; VCAM1; PVRL3; MPZL1; CDH1; CLDN8; CLDN1; CLDN3; SDC3* | 14/134 | 0.01 | 0.00 |
| | | B: *CD80; CD22; ITGA4* | | | |
| ECM-receptor interaction | 6 | E: *AGRN; SDC3; ITGA6; ITGB6; LAMC1; COL1A2; COL3A1; SPP1* | 9/84 | 0.02 | 0.02 |
| | | B: *ITGA4* | | | |
| Cytokine-cytokine receptor interaction | 6 | E: *CXCL14; CCL22; PRLR; INHBB; CCL24; CSF1R; PRL; LTBR; TNFRSF9; CCL8; CCL2; PDGFB* | 21/263 | 0.04 | 0.02 |
| | | B: *CCR3; IL8RB; IL18RAP; CRLF2; IFNAR1; TNFRSF1B; IL17RA; TNFRSF8; CLCF1* | | | |
| **Signal transduction** | | | | | |
| Phosphatidylinositol signaling system | 17 | E:*CLML5* | 7/76 | 0.08 | 0.00 |
| | | B: *CDS2; DGKH; DGKZ; INPP5K; PIK3R5; PIK3R3* | | | |
| TGF-beta signaling pathway | 7 | E: *MAPK3; INHBB; ID4; DCN* | 7/87 | 0.14 | 0.01 |
| | | B: *PPP2CA; CREBBP; E2F5* | | | |
| Jak-STAT signaling pathway | 7 | E: *BCL1L1; PRPL; CSH1; PRL* | 16/155 | 0.00 | 0.01 |
| | | B: *PIK3R5; CRFL2; IFNAR1; CLCF1; PIAS2; STAT5B; STAT1; PIK3R3; GRB2; CREBBP; STAT3; STAT5A* | | | |
| ErbB signaling pathway | 7 | E: *MAPK3; JUN; ERBB3* | 11/87 | 0.00 | 0.01 |
| | | B: *CDKN1A; MAP2K4; STAT5B; PIK3R5; PIK3R3; GRB2; STA5A; NCK1* | | | |
| Calcium signaling pathway | 7 | E: *CALM5; EDNRB; ERBB3; OXTR; SLC8A3* | 5/182 | 0.95 | 0.01 |

| | | | | | |
|---|---|---|---|---|---|
| | | B: none | | | |
| MAPK signaling pathway | 6 | E: *MAPK3; FOS; JUN; RAC3; PDGFB; NF1; RRAS; PLA2G3; FGF1; HSPA1B; HSPA1A; HSPA6* | 21/272 | 0.05 | 0.02 |
| | | B: *DUSP1; MAP4K2; MAP3K5; MAP3K8; GRB2; MAP2K3; RELA; MAP2K4; RASGRP4* | | | |
| Wnt signaling pathway | 5 | E: *PSEN1; FZD3; JUN; RAC3; WNT10B; DKK1* | 14/152 | 0.02 | 0.03 |
| | | B: *CSNK1A1; PPARD; CSNK1A1L; PPP2R5B; DAAM1; CREBBP; CSNK1E; PPP2CA* | | | |
| Cancers | | | | | |
| Pathways in cancer | 16 | E: *CDH1; ARNT2; PLD1; CSF1R; AR; CCNA1; FGF1; LAMC1; ITGA6; BCL2L1; WNT10B; PDGFD; RAC3; JUN; FOS; FZD3; EPAS1; MAPK3*<br>B: *CCNE1; PIK3R5; TRAF3; RARA; PIAS2; PML; STAT5B; STAT1; PIK3R3; PPARD; GRB2; RET; CDKN1A; RELA; CCNE2; CREBBP; STAT3; ARNT; RUNX1; STAT5A; TCEB1* | 39/330 | 0.00 | 0.00 |
| Acute myeloid leukemia | 13 | E: *CCNA1; MAPK3* | 13/59 | 0.00 | 0.00 |
| | | B: *STAT5A; RUNX1; STAT3; RELA; GRB2; PPARD; PIK3R3; STAT5B; PML; RARA; PIK3R5* | | | |
| Renal cell carcinoma | 9 | E*: MAPK3; JUN; PDGFB; EPAS1; ARNT2* | 11/69 | 0.00 | 0.00 |
| | | B: *PIK3R3; PIK3R5; GRB2; CREBBP; ARNT; TCEB1* | | | |
| Prostate cancer | 9 | E: *MAPK3; CREB5; PDGFB; CRE3L2; AR* | 13/90 | 0.00 | 0.00 |
| | | B: *PIK3R3; GRB2; CDKN1A; RELA; CCNE2; CREBBP* | | | |
| Chronic myeloid leukemia | 6 | E: *MAPK3; BCL2L1* | 10/75 | 0.00 | 0.01 |
| | | B: *PIK3R5; STAT5B; PIK3R3; GRB2; CDKN1A; RELA; RUNX1; STAT5A* | | | |
| Small cell lung cancer | 6 | E: *BCL2L1; ITGA6; LMAC1* | 10/86 | 0.01 | 0.02 |
| | | B: *CCNE1; PIK3R5; PIK3R3; TRAF3; PIAS2; RELA; CCNE2* | | | |
| Pancreatic cancer | 5 | E: *MAPK3; RAC3; BCL2L1; PLD1* | 9/72 | 0.01 | 0.03 |
| | | B: *PIK3R3; PIK3R5; STAT1; RELA; STAT3* | | | |
| Melanoma | 5 | E: *MAPK3; PDGFB; FGF-1; CDH1* | 7/71 | 0.05 | 0.05 |
| | | B: *PIK3R5; PIKR3R3; CDKN1A* | | | |
| Cell communication | | | | | |
| Adherens junction | 15 | E: *RAC3; LMO7; PVRL4; PVRL3; CDH1; MAPK3; PARD3;* | 10/78 | 0.01 | 0.00 |
| | | B: *CREBBP; FYN; SNAI1* | | | |
| Tight junction | 13 | E: *RRAS; AMOTL1; RAB13; CLDN3; CLDN1; CLDN8; CGN; PARD3* | 16/135 | 0.00 | 0.00 |
| | | B: *MYL2; EXOC4; ASHL1; MYLPF; MYH10; PPP2CA; CASK; PRKCI* | | | |
| Focal adhesion | 7 | E: *MAPK3; RAC3; PDGFB; JUN; ITGB6; ITGA6; LAMC1; COL1A2; COL3A1; SPP1* | 20/203 | 0.00 | 0.00 |
| | | B: *MYL2; PIK3R5; ITGA4; MYLPF; GRLF1; PXN; DIAPH1; GRB2; PIK3R3; FYN* | | | |
| Endocrine system | | | | | |
| PPAR signaling pathway | 8 | E: *NR1H3; PCK1; SCD; CYP27A1; CYP4A11; SLC27A2* | 11/70 | 0.00 | 0.00 |
| | | B: *CPT1A; CPT1C; OLR1; PPARD; GK* | | | |

| | | | | | |
|---|---|---|---|---|---|
| Renin-angiotensin system | 6 | E: *AGT; CTSA; MME*<br>B: none | 3/17 | 0.04 | 0.01 |
| Behavior | | | | | |
| Circadian rhythm | 10 | E: none<br>B: *CRY2; CSNK1E; PER1* | 3/13 | 0.02 | 0.00 |
| Infectious diseases | | | | | |
| Pathogenic Escherichia coli infection | 6 | E: *TUBB6; TUBA3C; KRT18; CLDN1; CDH1*<br>B: *FYN; NCK1* | 7/54 | 0.02 | 0.02 |
| Transcription | | | | | |
| Basal transcription factors | 5 | E: *GTF2A1; TAF1; GTF2I*<br>B: *TAF4B; TAF11; GTF2B* | 6/37 | 0.01 | 0.03 |
| Cell motility | | | | | |
| Regulation of actin cytoskeleton | 6 | E: *MAPK3; TMSB4X; RAC3; PDGFB; RRAS; ITGA6; ITGB6; ENAH; FGF1*<br>B*: MYL2; SSH2; PIK3R5; PIK3R3; ITGA4; GRLF1; MYLPF; MYH10; DIAPH1; PXN* | 19/217 | 0.01 | 0.02 |

[a]Pathway express was used to determine pathways that are differentially regulated.

[b]Abbreviations are as follows: IF, Impact Factor; IG/GP, number of differentially regulated genes/ total number of genes involved in the pathway; P, probability value by hypergeometric distribution analysis; γP: gamma P value, which is the P value corrected by impact factor.
