# Supplementary material for: Differentiation of the Endometrial Macrophage during Pregnancy in the Cow
Source: PLoS One. 2010 Oct 7;5(10):e13213. doi: 10.1371/journal.pone.0013213 (PMC2951363; doi:10.1371/journal.pone.0013213)
Supplement: Table S3 — Differentially regulated genes in endometrial and blood CD14+ cells that were also differentially regulated in the intermediately-differentiated, fully differentiated, M1 and M2 activated macrophage. (0.09 MB PDF) [file pone.0013213.s003.pdf]

Table S3. Differentially regulated genes in endometrial and blood CD14$^+$ cells that were also differentially regulated in the intermediately-differentiated, fully differentiated, M1 and M2 activated macrophage.[a]

Description	Accession	Intensity		Fold change	P	Tissue[b]
		Endometrium	Blood			
**Intermediately-differentiated macrophage**						
Upregulated genes during monocyte to macrophage differentiation						
Fc fragment of IgG, high affinity I, receptor for (*FCGR1*)	NM_174538	306	11	28	0.05	Endo
Chemokine (C-C motif) ligand 2 (*CCL2*)	NM_174006	18786	3526	5	0.01	Endo
Early growth response 1 (*EGR1*)	NM_001045875	64632	17902	4	0.02	Endo
Centromere protein A transcript variant 1 (*CENPA*)	XM_864817	11731	5692	2	0.02	Endo
Chromosome 6 open reading frame 115 (*C6orf115*)	XM_582278	10895	6280	2	0.02	Endo
Lectin, mannose-binding 2 (*LMAN2*)	NM_001101309	34142	17438	2	0.03	Endo
24-dehydrocholesterol reductase (*DHCR24*)	XM_613218	101952	47891	2	0.05	Endo
Opa interacting protein 5 (*OIP5*)	XM_588370	204	630	3	0.00	Blood
Downregulated genes during monocyte to macrophage differentiation						
Predicted: Palladin, cytoskeletal associated protein (*PALLD*)	XM_869983	753	48	16	0.00	Endo
BCL2-like 1, nuclear gene encoding mitochondrial protein (*BCL2L1*)	NM_001077486	2017	342	6	0.00	Endo
Inhibitor of growth family, member 3, transcript variant 2 (*ING3*)	XM_863966	4923	15733	3	0.05	Blood
**Fully-differentiated macrophage**						
Upregulated genes in differentiated macrophages						
Ribonuclease (*RNASE1*)	NM_001014386	5671	124	46	0.01	Endo
Folate receptor 2 (fetal) (*FOLR2*)	NM_001075325	1486	63	24	0.00	Endo
Component 1, q subcomponent, alpha polypeptide (*C1QA*)	NM_001014945	15126	793	19	0.00	Endo
Apolipoprotein E (*APOE*)	NM_173991	3576	208	17	0.00	Endo
Complement component 1, q subcomponent, B chain (*C1QB*)	NM_001046599	29681	2122	14	0.00	Endo
Transcobalamin II; macrocytic anemia (*TCN2*)	NM_174195	6560	811	8	0.03	Endo
Myloid beta (A4) precursor protein (*APP*)	NM_001076796	18442	2748	7	0.04	Endo
Sialoadhesin (*SIGLEC1*)	XM_870818	277	50	6	0.05	Endo
Hypothetical LOC539693 (*AK3L1*)	XM_588793	163	49	3	0.04	Endo
Phospholipase D family, member 3 (*PLD3*)	NM_001078041	3318	1003	3	0.03	Endo
Zinc finger, FYVE domain containing 26 (*ZFYVE26*)	XM_592247	6979	3169	2	0.02	Endo
Nuclear receptor subfamily 1, group H, member 3 (*NR1H3*)	NM_001014861	10998	5265	2	0.01	Endo
Scm-like with four MBT domains protein 2 (*SFMBT2*)	XM_607062	63	211	3	0.05	Blood
Downregulated genes in differentiated macrophages						
Desmoglein 2 preproprotein (*DSG2*)	XM_584890	201	7	30	0.05	Endo
Cystatin E/M (*CST6*)	NM_001012764	17864	1411	13	0.01	Endo

Ankycorbin (Retinoic acid-induced protein 14) (*RAI14*)	XM_604226	1960	160	12	0.00	Endo
Regulator of G-protein signaling 2, 24kDa (*RGS2*)	NM_001075596	57900	6307	9	0.05	Endo
Cytochrome P450, family 27, subfamily A, polypeptide 1 (*CYP27A1*)	NM_001083413	2018	366	6	0.04	Endo
FBJ murine osteosarcoma viral oncogene homolog (*FOS*)	NM_182786	34786	8393	4	0.02	Endo
Zinc finger, MYND domain containing 15 (predicted) (*ZMYND15*)	XM_589053	2419	868	3	0.02	Endo
DEAD (Asp-Glu-Ala-Asp) box polypeptide 17 (*DDX17*)	NM_001101993	41773	22419	2	0.03	Endo
Interleukin 27 receptor, alpha (*IL27RA*)	NM_001098028	28729	12407	2	0.05	Endo
Predicted: cytokine receptor-like factor 2 (*CRLF2*)	XM_608251	188	880	5	0.04	Blood
Low density lipoprotein (lectin-like) receptor 1 (*OLR1*)	NM_174132	564	2330	4	0.03	Blood
FYN oncogene related to SRC, FGR, YES (*FYN*)	NM_001077972	4343	7566	2	0.05	Blood
M1 vs M2 activation						
Overexpressed genes in M1 macrophages						
Complement factor B (*CFB*)	NM_001040526	18183	1647	11	0.03	Endo
Pentraxin-related gene, rapidly induced by IL-1 beta (*PTX3*)	NM_001076259	933	16716	18	0.05	Blood
Fas (TNF receptor superfamily, member 6) (*FAS*)	NM_174662	360	1971	5	0.03	Blood
Indoleamine 2,3-dioxygenase 1 (*IDO1*)	NM_001101866	380	1549	4	0.05	Blood
Overexpressed genes in M2 macrophages						
Solute carrier organic anion transporting polypeptide 2b1 (*SLCO2B1*)	NM_174843	8667	173	50	0.00	Endo
Glycine amidinotransferase (L-arginine:glycine amidinotransferase) (*GATM*)	NM_001045878	7387	240	31	0.00	Endo
Predicted: mannose receptor C1 (MRC1)	XM_001252128	2246	93	24	0.00	Endo
Aldehyde dehydrogenase 1 family, member A1 (*ALDH1A1*)	NM_174239	3722	210	18	0.01	Endo
Prostaglandin-endoperoxide synthase 1 (prostaglandin G/H synthase and cyclooxygenase 1) (*PTGS1*)	NM_001105323	2102	242	9	0.03	Endo
Ribonuclease, RNase A family, k6 (*RNASE6*)	NM_174594	33082	4824	7	0.04	Endo
C-type lectin domain family 7, member A (*CLEC7A*)	NM_001031852	50	22	2	0.03	Endo
Predicted: dipeptidase 2 (*DPEP2*)	XM_586714	140	328	2	0.05	Blood

[a]The list of genes that are differentially regulated was obtained from reference 23.

[b]Tissue with higher expression in the current experiment. Endo signifies that expression was higher for endometrial $CD14^+$ cells whereas Blood signifies higher expression in blood $CD14^+$ cells.
